# The adsorption characteristics and degradation mechanism of tinidazole on an anatase TiO_2_ surface: a DFT study†

**DOI:** 10.1039/c9ra06665a

**Published:** 2020-01-10

**Authors:** Qiaoqiao Qin, Haichuan Qin, Kai Li, Ruolan Tan, Xiangyang Liu, Laicai Li

**Affiliations:** College of Chemistry and Material Science, Sichuan Normal University Chengdu 610068 China lilcmail@163.com; College of Pharmacy, Southwestern Medical University Luzhou 646000 China

## Abstract

The adsorption characteristics and degradation mechanism of tinidazole on TiO_2_(101) and (001) surfaces under vacuum and aqueous solution conditions were studied by density functional theory (DFT). The results show that tinidazole can adsorb on the surfaces of TiO_2_(101) and (001) under different conditions. The hydrogen bond generated during the adsorption process can enhance the stability of the adsorption configuration, which makes the bond length of C–N of tinidazole longer and finally facilitates the ring-opening degradation reaction. As for the mechanism of the ring-opening degradation reaction, it was found that ring-opening can be carried out along reaction route II on both crystal surfaces, and the reaction activation energy is lower on (101) surface. Under the conditions of aqueous solution, the decrease of the activation energy of the ring-opening degradation reaction indicates that the solvent conditions can promote the degradation reaction.

## Abstract 2

The adsorption characteristics and degradation mechanism of tinidazole on TiO_2_(101) and (001) surfaces under vacuum and aqueous solution conditions were studied by density functional theory (DFT).

## Graphical abstract

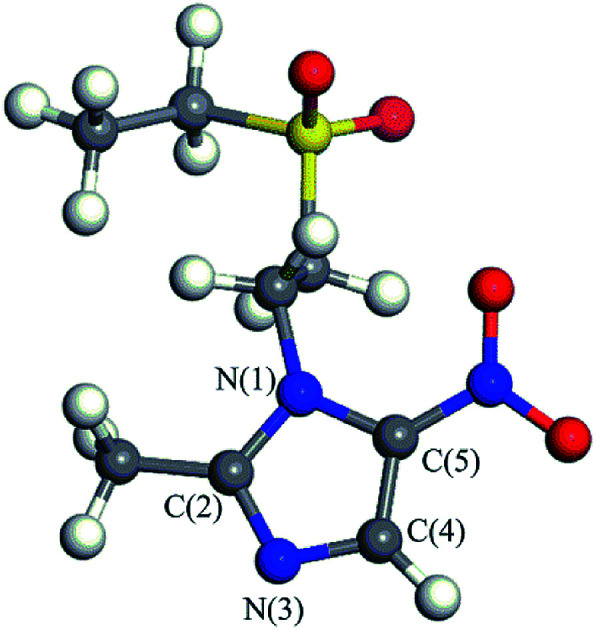

## 1. Introduction

**Fig. 1:**
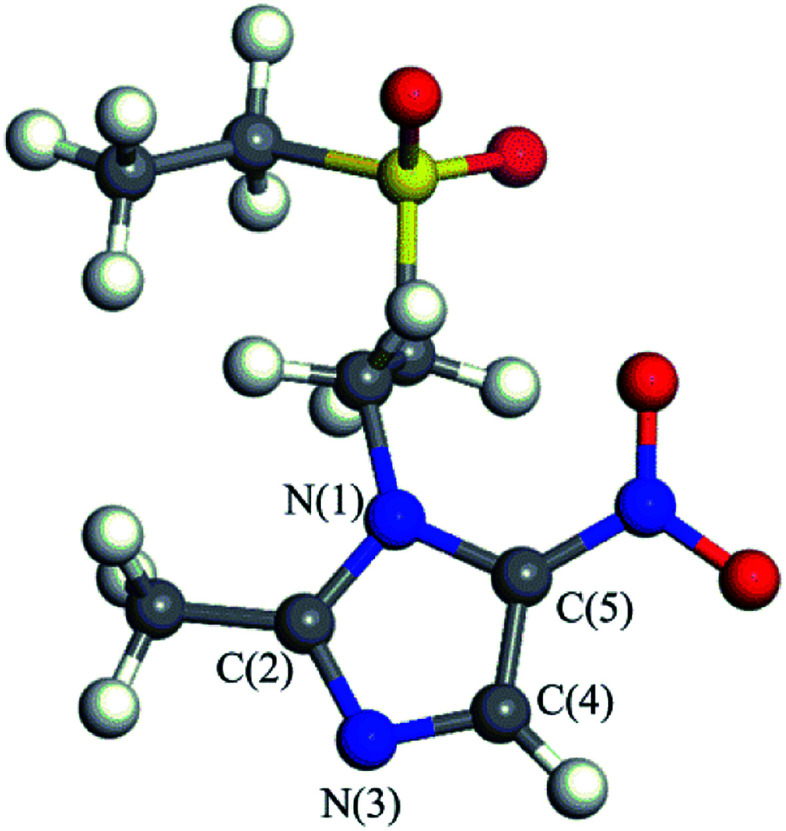
The molecular structure of tinidazole (TNZ).

Tinidazole (TNZ, chemical structure shown in Fig. 1) is a second-generation nitroimidazole antibiotic^1^ with antibacterial and anti-inflammatory effects. It has been widely used to prevent and treat the infection of amoeba, vaginal trichomoniasis, giardiasis, and also used as a growth promoter in animal husbandry and aquaculture.^2–4^ However, with the widespread use of tinidazole and lack of suitable regulation, environmental problems have further increased. The presence of tinidazole has been detected in some wastewater treatment plants and freshwater systems.^5^ Tinidazole that remains in water, even at low concentrations, can pose a long term potential threat to humans and the environment.^6^ Therefore, how to remove tinidazole effectively from the environment is an urgent problem to be solved. Compared with the studies on the degradation of tinidazole, there are many methods for the degradation of other nitroimidazoles, such as adsorption, biodegradation, Fenton method, photocatalysis, *etc.* The adsorption method is widely used for the treatment of organic waste water. As an example, moral-Rodriguez's work indicates that the ronidazole (RNZ) can be adsorbed on granular activated carbon (GAC) *via* π–π interactions.^7^ However, this method does not really remove the contaminant, only transfers the contaminant from the aqueous phase to the solid phase.^8^ The biological method is another common method, but it is generally time consuming, and the degradation is influenced by many factors, and the degradation effect is very different.^9^ Alexy *et al.*^10^ conducted a closed bottle experiment on 18 antibiotics, and found that metronidazole only degraded up to 1% after many days, indicating that the nitroimidazoles were hardly degraded by traditional biological method. The Fenton method refers to that under acidic conditions, Fe^2+^ and H_2_O_2_ combine to produce ·OH radicals, and then ·OH radicals convert refractory substances into small molecules or directly convert into CO_2_, H_2_O and some inorganic ions.^11,12^ Xiong^13^ used Fenton, UV/Fenton methods to degrade the low concentration of metronidazole in water system, the results show that the removal rate of the remains are 66.6% and 95.8%, respectively. Although the Fenton and UV/Fenton methods have higher degradation efficiency of metronidazole, the precipitation of Fe(OH)_3_ in the degradation process may also become a new pollutant, and the degradation efficiency is greatly affected by the concentration and the feed ratio of Fe^2+^ and H_2_O_2_. Photocatalysis is a process of mineralizing these contaminants into CO_2_, H_2_O and other inorganic substances by generating strong oxidizing OH radicals.^14–16^ It is considered to be a very effective method for the treatment of nitroimidazole residues.^17^ Metal oxides or sulfides are often used as semiconductor photocatalysts to degrade residual contaminants,^18^ such as TiO_2_, ZnO, GaO, CdS, ZnS. Among them, TiO_2_, ZnO and CdS have the strongest activity, but ZnO and CdS have low photostability and they are prone to photocorrosion, producing biotoxic Zn^2+^, Cd^2+^.^19,20^ Therefore, TiO_2_ is the most widely used semiconductor material in photocatalysis, due to its high photocatalytic activity, chemical stability, low cost, and without introducing secondary pollutant. Farzadkia *et al.*^21^ used UV/TiO_2_ to degrade metronidazole with a high removal rate, and biodegradable intermediates were formed during the process of photocatalytic degradation. Photocatalytic method can achieve similar degradation effect on dimetridazole. Chen^22^ degraded dimetridazole with TiO_2_ as photocatalyst, which can achieve high removal rate. To investigate the mechanism of these reactions, many theoretical works have been conducted. For instance, we have studied the adsorption characteristics and degradation mechanism of metronidazole and dimetridazole.^23–25^ Tinidazole, similar to metronidazole and dimetridazole, is also one kind of nitroimidazole antibiotics and TiO_2_ photocatalysis can therefore be used for the degradation of tinidazole as its use in the degradation of metronidazole and dimetridazole. Umar *et al.*'s^26^ experimental research indicate that TiO_2_ can efficiently catalyze the photomineralization of organic pollutants tinidazole in the presence of hydrogen peroxide, light and atmospheric oxygen. However, the underlying mechanism of TiO_2_ catalyzed degradation of tinidazole remains to be explored, which always need the help of the high level theoretical calculations. Motivated by this, in this paper, we investigated the adsorption characteristics and the ring-opening degradation reaction of tinidazole on TiO_2_(101) and (001) surfaces under vacuum and aqueous conditions.

## 2. Methodology

**Fig. 2:**
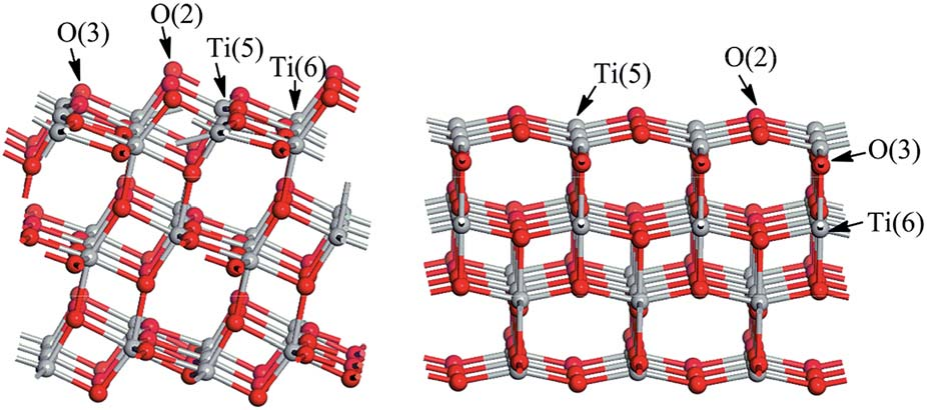
TiO_2_(101) and (001) surface models.

There are two kinds of Ti atoms and two kinds of O atoms (Fig. 2). Based on a preliminary study of the effect of plate thickness on surface energy, when the (101) surface adopts a three-layer model^27^ and the (001) surface adopts a single layer model,^28^ the balance between calculation time and accuracy can be achieved. To avoid the interactions between the periodic images, a 15 Å vacuum region along *Z* direction was added. The (3 × 1) supercell and (3 × 3) supercell was used for anatase TiO_2_(101) and (001) surfaces respectively with (TiO_2_)_36_ composition. The corresponding surface areas are 10.89 Å × 11.33 Å and 11.33 Å × 11.33 Å on the (101) and (001) surfaces of TiO_2_, respectively. In order to simulate the effect of solvent, we add about 48 and 69 H_2_O molecules with the density of 1 g cm^−3^ on the TiO_2_(101) and (001) surfaces respectively. These water molecules are described by the universal force field (UFF).^29^

The tinidazole molecule was placed above the TiO_2_(101) and (001) surfaces. The distance between the tinidazole molecule and the TiO_2_ surfaces were set to about 3.8 Å to eliminate the strong interaction between tinidazole molecule and TiO_2_ surface. Then we adopted the ReaxFF force field for molecular dynamics simulations.^30^ The system is first heated to 300 K with repeated velocity rescaling methods and then followed by a 10 ps NVE molecular simulations.^31^ The time step and the total simulation time are 0.1 fs and 10 ps respectively. The local minima of the NVE simulations were then taken as the initial structures for the following DFT calculations. All the molecular dynamics simulations are carried out by the LAMMPS program.

All DFT calculations are based on periodic boundary conditions,^32^ with the generalized gradient approximation (GGA) to the electronic exchange electron exchange and correlation energy, using the Perdew–Burke–Ernzerhof (PBE) functional.^33^ In our present work, the Brillouin-zone integrations were calculated using Monkhorst–Pack grids of special points. A 3 × 3 × 1 *k*-point grid was used in our DFT calculations. The electrons use effective core potentials (ECP) to describe,^34^ and the atomic orbital basis set is described by a polarizable DNP^35^ basis set with a cutoff radius of 4.5 Å. The transition state is searched by LST/QST^36^ method and confirmed by frequency analysis. The solvation effect of the reaction is studied by using COSMO model^37,38^ with water as solvent. In this paper, the self-consistent iterative energy (SCF) convergence criterion is 1.0 × 10^−5^ Ha, while the geometry convergence were judged by several conditions: the force is less than 0.004 Ha Å^−1^, the maximum displacement is 5 × 10^−3^ Å, and the energy convergence criterion is 2.0 × 10^−5^ Ha. All the DFT calculations are conducted by the Dmol^3^ module implemented in Materials Studio.

The adsorption energy in the calculation process is defined as the change of material energy before and after adsorption.*E*_ads_ = (*E* + *E*_sur_) − *E*_adsorption_*E*_adsorption_ is the total energy of the adsorbed system, *E* is the energy of the adsorbed material, and *E*_sur_ is the energy of the adsorbed surface.

The energy gap is the difference between the valence band maximum (VBM) and the conduction band minimum (CBM) energies.*E*_gap_ = *E*_CBM_ − *E*_VBM_

The lattice parameters of the bulk anatase TiO_2_ optimized by the above method are: *a* = *b* = 3.776 Å, *c* = 9.486 Å, which is in good agreement with previous works: *a* = *b* = 3.785 Å, *c* = 9.514 Å,^39–41^ indicating that our calculation method is reliable.

## 3. Results and discussion

**Fig. 3:**
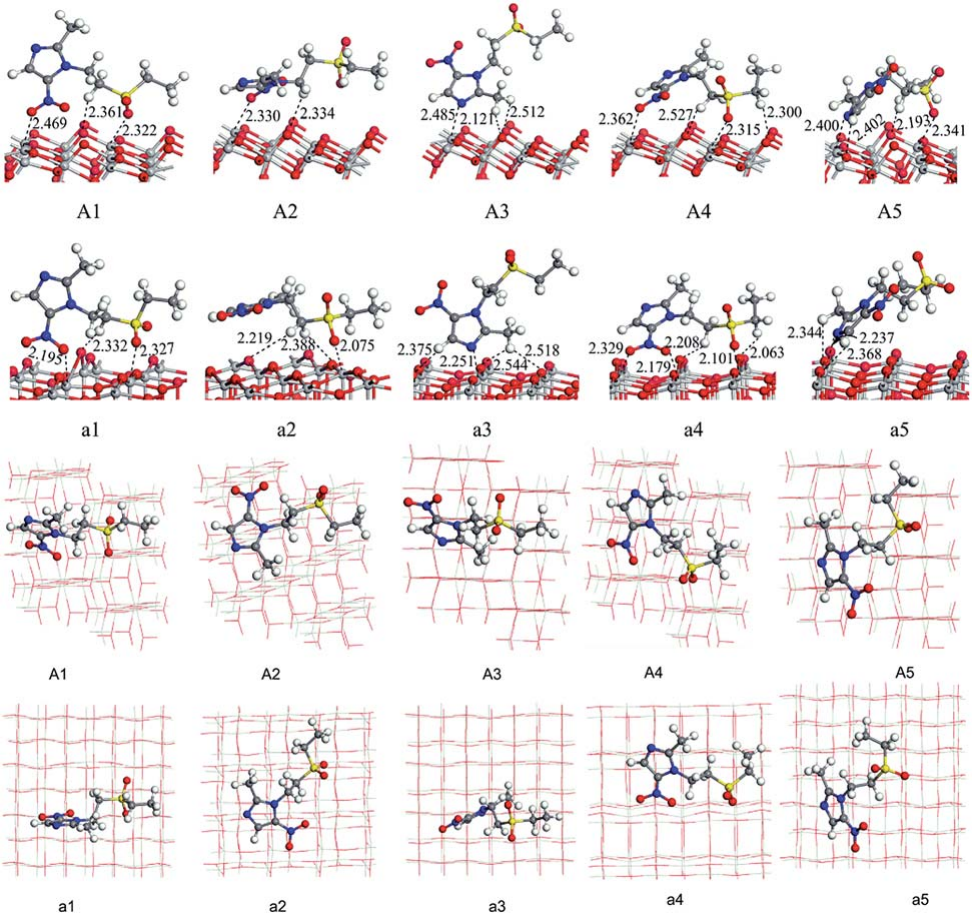
Adsorption configurations and adsorption distances (Å) of tinidazole on TiO_2_(101) and (001) surfaces under vacuum conditions (front view and top view).

We have optimized the molecular structure of tinidazole (Fig. S1, ESI†) and the stable surfaces of TiO_2_(101) and (001), and the selected local minima of adsorption configurations in previous molecular dynamics simulations. The calculated adsorption energies and energy gaps of these adsorption configurations are listed in Table 1. A1–A5 and a1–a5 are respectively five stable configurations of tinidazole adsorbed on the TiO_2_(101) and (001) surfaces under vacuum conditions (Fig. 3). The nitro moiety O atom on the imidazole ring and the sulfonyl group O atom can be adsorbed on the Ti(5) atom on the surface of TiO_2_. The H atom of the C(2) methyl group and the H atom on the N(1) branch, and the H atom on C(4) can form hydrogen bonds with the O(2) atom of the TiO_2_ surface. Li^42^ studied the catechol/TiO_2_ interface adsorption and found that hydrogen bond can lower the reaction energy barrier and increase the stability of adsorption configuration. Chang's^43^ works revealed that the hydrogen bond between HNO_3_ and TiO_2_ can also enhance the stability of adsorption configuration. According to their results, the most stable molecular configuration corresponds to the situation in which the O atom of nitric acid attached to the Ti_5c_ atom on the surface while the hydrogen atom formed a hydrogen bond with the nearest bridge oxygen on the surface. All these works indicate the formation of hydrogen bonds can increase the stability of the adsorption configuration.

**Table 1:** Adsorption energies and energy gap values for adsorption configurations of tinidazole adsorbed on TiO_2_ (101) and (001) surfaces

Conditions	(101) surface			(001) surface		
	Compound	*E*_ads_ (eV)	Energy gap (eV)	Compound	*E*_ads_ (eV)	Energy gap (eV)
Vacuum conditions	A1	0.71	2.47	a1	1.80	1.46
	A2	0.51	2.09	a2	1.79	1.91
	A3	0.66	2.05	a3	1.87	1.63
	A4	0.75	2.46	a4	1.73	1.55
	A5	0.89	2.45	a5	2.07	2.11
Aqueous solutions	B1	2.44	2.27	b1	2.58	1.96
	B2	2.22	2.18	b2	2.61	1.91
	B3	1.90	2.34	b3	2.51	1.87
	B4	2.31	2.37	b4	1.81	1.56
	B5	2.22	2.26	b5	2.11	1.90

Among all adsorption configurations of tinidazole on the TiO_2_(101) surface under vacuum conditions (Table 1), A5 is the most stable one with the highest adsorption energy of 0.89 eV. In A5 configuration, the N(3) atom on the imidazole ring is adsorbed the Ti(5) atom on the TiO_2_(101) surface, the adsorption distance is 2.400 Å. The H atoms of the C(4) atom and the C(2) atom form hydrogen bonds with the O(2) atom on the TiO_2_(101) surface, and the hydrogen bonds are 2.402 and 2.422 Å, respectively. Due to the interactions with the surface of TiO_2_, some bond lengths of tinidazole molecule have also changed. C(2)–N(1) and N(3)–C(4) on the imidazole ring were changed from 1.335 and 1.354 Å before adsorption to 1.352 and 1.361 Å, respectively, and ∠C(2)–N(3)–N(4) was changed from 106.1° to 107.3° after adsorption. The C(2)–N(1) bond elongation is greater than the N(3)–C(4) bond, which indicates that the adsorption process makes C(2)–N(1) more unstable. Some studies have shown that the degradation of nitrogen heterocyclic compounds generally begins with the carbonization process adjacent to the nitrogen atom,^44^ and the weakening of the C(2)–N(1) bond is beneficial to the attack of hydroxyl radicals. There is no case that the N(3) atom adsorbed on the Ti(6) atom and the H atom adsorbed on O(3) atom in the A1 to A5 structures, which indicates that Ti(5) and O(2) atoms are more active than the Ti(6) and O(3) atoms.

On the (001) surface, under vacuum conditions, the adsorption energy of a5 (2.07 eV) is the largest (Table 1), which is the most stable adsorption configuration. In a5 configuration, the N(3) atom is adsorbed on the Ti(5) atom, the adsorption distance is 2.237 Å. The H atom on the C(2) atom and the H atom of the C(4) methyl group are adsorbed on O atoms of TiO_2_(001) surface, the adsorption distances are 2.344 and 2.368 Å, respectively. Compared with the structure before the adsorption of tinidazole, the bond length of C(2)–N(1) and N(3)–C(4) increase from the original 1.322 and 1.356 Å to 1.357 and 1.364 Å, respectively due to the interaction between the N(3) atom of tinidazole and the Ti(5) atom on the surface of TiO_2_(001). The partial bond length of tinidazole is activated, and the weakening of N(1)–C(2) bond is beneficial to the attack of hydroxyl radical.

In order to consider the adsorption characteristics of tinidazole molecules on the TiO_2_ surface under aqueous conditions, we optimized the stable adsorption structures of tinidazole on TiO_2_(101) and (001) surfaces with some water molecules above the surface. B1–B5 and b1–b5 are five stable adsorption configurations of tinidazole on TiO_2_(101) and (001) surfaces considering the involvement of water molecules (Fig. S1, ESI†). Due to the addition of the water solvent model, the H atoms of H_2_O molecules interact with the O atoms on the TiO_2_ surface to form hydrogen bonds. The O atoms of the H_2_O molecules and Ti atoms form Ti–O bonds on the (101) surface, forming Ti–O⋯H bonds on the (001) surface. The adsorption energy of B1 (2.44 eV) is the largest, which is the most stable adsorption configuration (Table 1). In B1 structure, the sulfonyl O atom of the tinidazole is adsorbed on the Ti(5) atom of the TiO_2_(101) surface, and the adsorption distance is 2.291 Å. Meanwhile, the H atom on the N(1) branch of tinidazole and the O(2) atom on TiO_2_(101) surface forms a hydrogen bond with a bond length of 2.523 Å. The adsorption increased the bond lengths of N(1)–C(2) and N(3)–C(4) from 1.322 and 1.357 Å to 1.347 and 1.359 Å, respectively. The analysis of the bond lengths of the five configurations revealed that the N(1)–C(2) bond length increased significantly, indicating that under the action of water molecules, this bond became weak and susceptible to attacked by hydroxyl radicals. As for in the cases of adsorption in TiO_2_(001) surface, the b2 adsorption structure is the optimal adsorption configuration with the largest adsorption energy of about 2.61 eV, compared with the structure before adsorption of tinidazole, the bond lengths of N(1)–C(2) and N(3)–C(4) of the b2 structure increase from 1.322 and 1.357 Å to 1.343 and 1.358 Å, respectively. In aqueous solution, the most stable configurations are B1 and b2 on TiO_2_(101) and (001) surfaces, respectively. At the same time, adsorption makes the bond length of N(1)–C(2) of tinidazole becomes longer relative to the gas phase. In the water solvent adsorption model, H_2_O molecules are distributed around tinidazole, and there may be a strong interaction between the H_2_O molecules and the tinidazole molecule, so that the adsorption energy has increased compared to vacuum condition. Zhang^45^*et al.* studied the adsorption of CO molecule on CuCl(111) surface in solvent condition. Mendive^46^ studied the adsorption of oxalate on anatase(100) and rutile(110) in aqueous solution. These studies and our results illustrate that the water solvent could stabilize the adsorption on the surface.

In order to further investigate the interaction and bonding characteristics of tinidazole molecules with TiO_2_ surface, we calculated the density of states of tinidazole molecule on the TiO_2_ surface under vacuum conditions. In its band structure, the valence band is mainly composed of the 2p orbital of O atom of TiO_2_ and the conduction band is mainly composed of the 3d orbital of Ti atom of TiO_2_. Before TiO_2_ adsorption, the energy gap is to be 2.87 eV, which is close to the experimental value of 3.20 eV.^47^ After adsorption, the valence band and conduction band energy levels of TiO_2_ decreased, the energy gap became narrow. We found that the energy gap of TiO_2_ in A1–A5 is narrowed to 2.47, 2.09, 2.05, 2.46 and 2.45 eV after adsorption. In a1–a5, the energy gap of TiO_2_ is reduced to 1.46, 1.91, 1.63, 1.55 and 2.11 eV. Except for a1 and a4, all in the visible light range (the visible light energy gap is about 1.59–3.26 eV),^48^ which indicates that the visible light can drive the degradation of tinidazole on the surface of TiO_2_(Fig. S2 and S3, ESI†).

The band structure of TiO_2_ changes due to the H_2_O molecules on the surface of TiO_2_. After adsorption, the Ti 3d states still govern the conduction band edge, but the s orbital component of the valence band energy level is increased. Two peaks of the s orbital between −21 eV and −15 eV has formed, and the energy range of the p orbital is broadened from −5–0 eV to −8–0 eV. From −8–0 eV, two sets of peaks appear in which the peak height and peak area are increased compared with that of pure TiO_2_. The increase of the s and p orbital components indicates that the 1s orbital of the H atom and the 2p orbital of the O atom in the H_2_O molecules participate in hybridization. The s orbital appears near the Fermi level, which corresponds to the VBM of the system. When the number of electrons near the VBM increases, the electron donating ability of the system also increases, indicating that the chemical activity of TiO_2_ is enhanced. It is also found that the TiO_2_(001) surface has more p-orbital components, indicates that there may be more 2p orbitals of O in the H_2_O molecule participating in the hybridization, and the chemical activity of the TiO_2_(001) surface is more enhanced. In the photocatalytic reaction, H_2_O is essential. The TiO_2_ energy gap is narrowed after adsorption. The energy gap of B1–B5 are reduced to 2.27, 2.18, 2.34, 2.37 and 2.26 eV, the light absorption frequency is reduced and all in the visible wavelength range. The b1–b5 energy gap is reduced to 1.96, 1.91, 1.87, 1.56 and 1.90 eV, respectively, except b4 all in the visible light range. This indicates that the TiO_2_(001) surface can effectively utilize visible light under water solvent conditions (Fig. S4 and S5, ESI†).

**Scheme 1:**
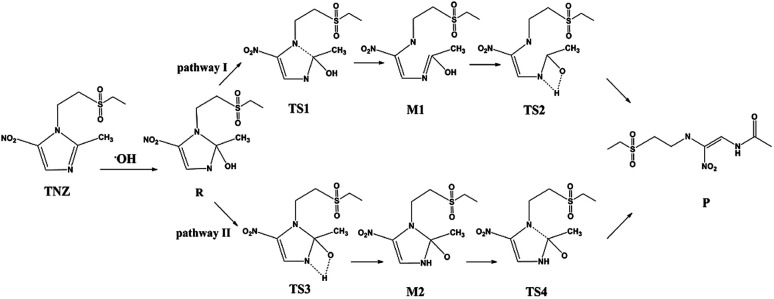
The ring-opening reaction mechanism of tinidazole.

When the TiO_2_ photocatalyst is irradiated by photons larger than its forbidden bandwidth, it is excited to generate photogenerated electron–hole pairs. The electrons have strong reducibility, and the holes have strong oxidizing properties. Some electrons and holes separate and migrate to the surface of the photocatalyst. The O_2_ adsorbed on the surface of TiO_2_ is reduced to superoxide anion (O_2_^−^), and the holes oxidize H_2_O adsorbed on the catalyst to ·OH radicals, and the antibiotic molecules are degraded by active substances O_2_^−^ and ·OH.^49–51^ Umar *et al.*^26^ concluded that TiO_2_ can efficiently catalyse the photomineralization of organic tinidazole in the presence of light and atmospheric oxygen, which is consistent with our research results. The ring opening of imidazole ring is a key step for antibiotic degradation, which contains two steps: C–N bond break and proton transfer on the imidazole ring.^26,52^ The reaction on the imidazole ring is generally considered to be a ring opening degradation reaction, mainly caused by a nucleophilic attack at the C2 position of the imidazole ring. To explore the ring-opening reaction of tinidazole on the TiO_2_(101) and (001) surfaces, we designed two reaction pathways and also studied the solvation effect (Scheme 1). Starting from the stable configuration in the adsorption study, the most stable A5 and a5 were selected as the starting materials for the ring-opening reaction. After attacked by the surface ·OH radicals, the starting materials are converted into co-adsorbed matter R and R*. The formation of intermediates R and R*, the C(2)–N(1) bond grows to the greatest extent, so we think that the ring-open degradation site is at the break of C(2)–N(1) on the imidazole ring. Reaction pathway I, first C(2)–N(1) is broken, then the H(1) atom on the hydroxyl group is transferred to the N(3) atom of the imidazole ring, and an enol structure is formed at the C(2) atom. When the H(1) atom is directly transferred to the N(3) atom, it will through a transition state of a four-membered ring structure, and finally form a product. In the reaction route II, the H(1) atom on the hydroxyl group is transferred to the N(3) atom of the imidazole ring through a four-membered ring transition state, and then the C(2)–N(1) bond is broken to form a product.

**Fig. 4:**
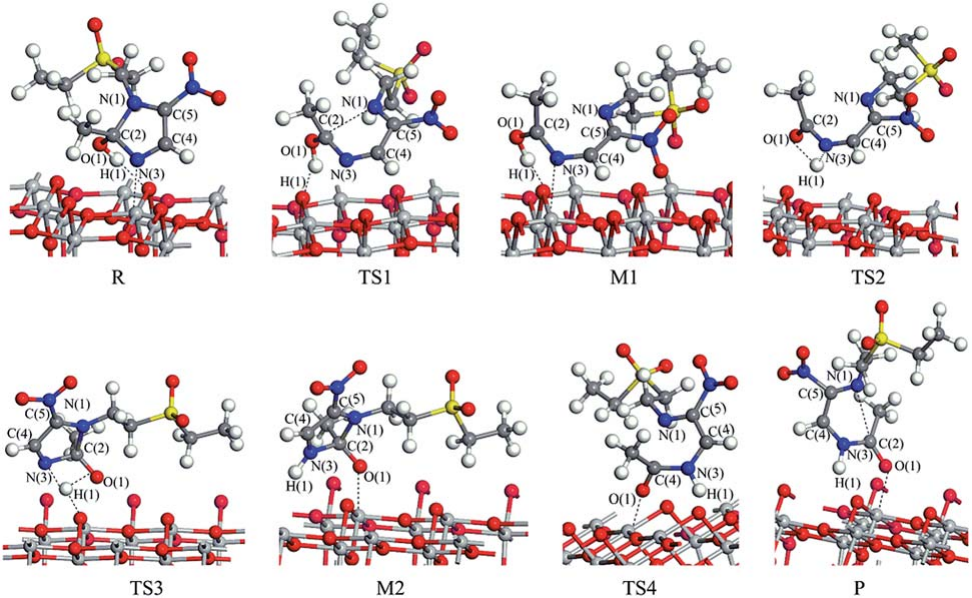
The configuration of substances in the reaction of tinidazole on the TiO_2_(101) surface in vacuum conditions (front view).

The reaction pathway I is R → TS1 → M1 → TS2 → P for the TiO_2_(101) surface. The front view is shown in Fig. 4, and the top view is in the ESI of Fig. S6.† In the starting material R, the tinidazole molecule is adsorbed on TiO_2_, the distance between N(3) and Ti(5) atoms is 2.298 Å. Meanwhile, ·OH radical attacks C(2) atom, C(2)–O(1) bond length is 1.388 Å, and the distance between H(1) and O(2) atoms is 1.752 Å. C(2)–N(1), N(3)–C(2) bond lengths are extended to 1.525 and 1.470 Å, respectively. The bond lengths of C(2)–N(1) and N(3)–C(2) is significantly longer than other covalent bonds on the imidazole ring, indicating that they may not be as strong as the other bonds. While the bond length of C(2)–N(1) is slightly longer than the N(3)–C(2) bond, so from the perspective of bond length, tinidazole has the highest probability of C(2)–N(1) cleavage and ring opening under the action of ·OH radicals. In transition state TS1, N(1)–C(2) begins to break and the bond length is extended to 2.345 Å. The activation energy of R1 → TS1 is 28.63 kcal mol^−1^, with a unique imaginary frequency of −260 i cm^−1^, and the direction of vibration is correctly directed to the reactants and products. In M1, N(1)–C(2) is completely broken and the distance between them is 2.944 Å. The recovery of double bond between C(2) and N(3) atoms and the formation of an enol form at C(2) atom structure. In TS2, H(1) atom is transferred from O(1) to N(3) atom, forming a four-membered ring transition state. The TS2 has a unique imaginary frequency of −1141 i cm^−1^, due to TS2 is far from the surface of TiO_2_, the interaction is weak, and the activation energy is as high as 51.57 kcal mol^−1^, which makes the reaction difficult to carry out. However, the activation energy of the process of R → TS1 → M1 is not high, and in this process the imidazole ring has ring-opened, indicating that ring-opening degradation reaction can occur. In P, the H(1) transfers to and bonds with N(3), the bond length of N(3)–H(1) is 1.053 Å, the N(3)–C(2) become single bond again.

The mechanism of reaction pathway II is R → TS3 → M2 → TS4 → P. From the process of R → TS3, the H(1) atom begins to separate from the O(1) atom and transfer to the N(3) atom, and a coplanar transition state of the four-membered ring is formed. The activation energy of TS3 is 21.44 kcal mol^−1^, with a unique imaginary frequency is −1646 i cm^−1^, and the direction of vibration is correctly directed to the reactants and products. H(1) atom has transferred to the N(3) atom, and the H(1)–N(3) bond length is 1.042 Å in M2 (Table S1, ESI†). The distances between Ti(5) and O(1), H(1) and O(2) are 2.044 and 1.961 Å, respectively. It is worth noting that the bond lengths of C(2)–N(3) and C(2)–N(1) in M2 are very long compared to tinidazole molecule, reaching 1.520 and 1.552 respectively. In addition, the O(1) atom loses a proton, and the O(1)–C(2) bond length is shortened to 1.323 Å, which has a tendency to form a double bond. In TS4, the C(2)–N(1) bond starts to break, and the bond length is extended to 2.201 Å. The N(3)–C(2) and C(2)–O(1) bond lengths are shortened to 1.462 and 1.240 Å, respectively. It indicating that the covalent bond between N(3) and C(2) atoms and the double bond between C(2) and O(1) atoms are gradually formed. The H(1) and O(1) atoms are adsorbed on the surface of TiO_2_, the distances between H(1) and O(2), O(1) and Ti(5) are 1.843 and 2.128 Å, respectively. The activation energy of TS4 is 13.84 kcal mol^−1^, with a unique imaginary frequency is −129 i cm^−1^, and the vibration direction is correctly directed to the reactants and products. Finally, P is formed by TS4, C(2)–N(1) is completely broken, and the distance between them is 3.206 Å. Meanwhile, C(2)–N(3) and C(2)–O(1) are also completely formed, the bond lengths are 1.372 and 1.258 Å.

The reaction mechanism of tinidazole on the TiO_2_(001) surface is the same to the (101) surface. The reaction route I: R* → TS1* → M1* → TS2* → P*. In R*, the tinidazole molecule is adsorbed on the TiO_2_ surface, and the distance between N(3) and Ti(5) atoms is 2.519 Å. Meanwhile, ·OH radical attacks the C(2) atom, the C(2)–O(1) bond length is 1.397 Å, and the distance between H(1) atom and O(2) atom is 1.851 Å. The C(2)–N(1) bond length is increased from the original 1.370 Å to 1.519 Å (Table S3, ESI†). The double bond N(3)–C(2) becomes a single bond, and the bond length is extended from the original 1.332 Å to 1.473 Å, the C(2) atom is sp^3^ hybrid. The bond length of N(3)–C(2) and C(2)–N(1) are significantly longer than the other covalent bonds on the imidazole ring, so from the perspective of the bond length, the two bonds may not be as strong as the other bonds. The C(2)–N(1) bond is slightly longer than the N(3)–C(2) bond, so C(2)–N(1) bond is the most likely to ring opening under the action of ·OH radicals. In the transition state TS1*, N(1)–C(2) bond begins to break, and the bond length is further extended to 2.199 Å. The activation energy of R1* → TS1* is 20.70 kcal mol^−1^, and there is a unique imaginary frequency of −264 i cm^−1^, and the vibration direction is correctly point to the reactants and products. In M1*, the N(1)–C(2) bond is completely broken, the distance between them is 3.043 Å. The bond between C(2) and N(3) atoms are restored with a bond length of 1.330 Å, and an enol structure is formed at the C(2) atom. In the transition state TS2*, H(1) atom is transferred from O(1) to N(3) atom to form a four-membered ring transition state. Since TS2* is far from the surface of TiO_2_, the interaction between them is weak, and the activation energy is as high as 58.02 kcal mol^−1^, which makes the reaction is difficult to carry out. However, the process of R* → TS1* → M1*, the imidazole ring has opened and the degradation purpose is initially achieve. The transition state TS2* has a unique imaginary frequency of −1549 i cm^−1^, and the vibration direction is correctly directed to the reactant and product. In P*, H(1) atom has been transferred to the N(3) atom, and the N(3)–H(1) bond length is 1.046 Å. N(3) and C(2) atoms become single bond again, and the double bond between C(2) and O(1) atoms form a bond length of 1.248 Å. At this time, the distance between N(1) and C(2) atoms is 3.177 Å. O(1) adsorption on the surface of TiO_2_, the distance between the two is 2.289 Å. At the same time, H(1) is also adsorbed to the surface of TiO_2_, and the distance between H(1) and the O(2) atom is 1.998 Å.

**Fig. 5:**
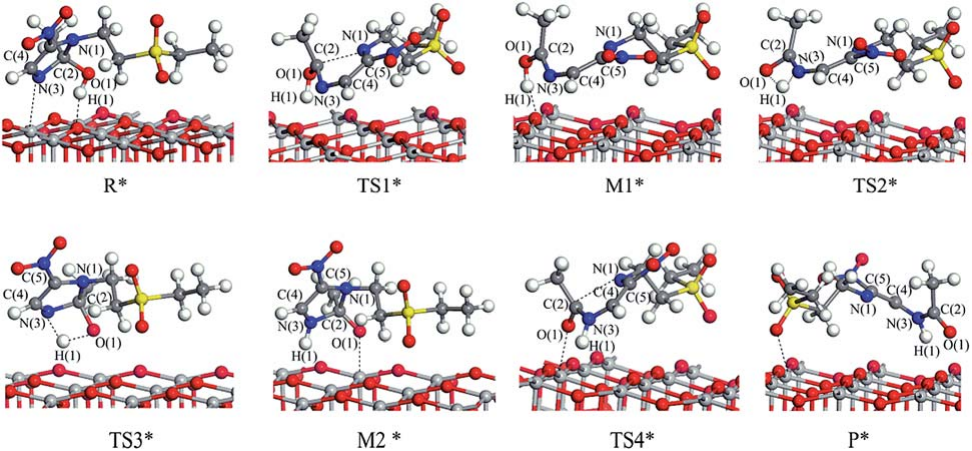
The configuration of substances in the reaction of tinidazole on the TiO_2_(001) surface in vacuum conditions (front view).

**Fig. 6:**
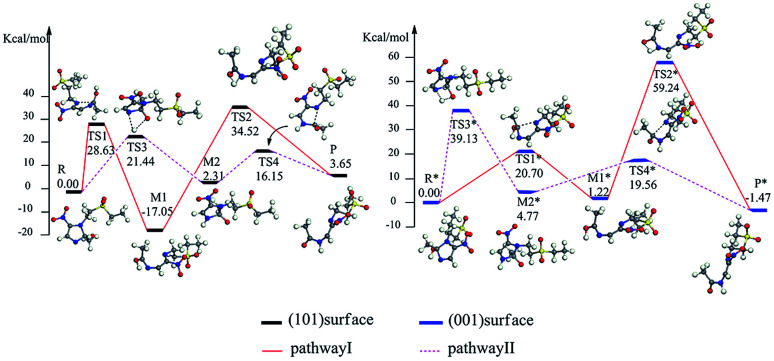
Energy level of tinidazole on the surfaces of TiO_2_(101) and (001) in the reaction (kcal mol^−1^).

Reaction route II is the optimal reaction pathway for tinidazole to open-loop reaction on TiO_2_(001) surface, which is R* → TS3* → M2* → TS4* → P*. The front view is shown in Fig. 5, and the top view is in the ESI of Fig. S7.† From R* → TS3*, the H(1) atom begins to separate from the O(1) atom and transfer to the N(3) atom to form a four-membered ring transition state. The activation energy of the transition state TS3* is 39.13 kcal mol^−1^, with a unique imaginary frequency of −1265 i cm^−1^, and the direction of vibration is correctly directed to the reactants and products. In M2*, the H(1) atom has been transferred to the N(3) atom, and the bond length of H(1)–N(3) is 1.035 Å. O(1) and H(1) atoms are attracted to the surface of TiO_2_, the distance between O(1) and Ti(5), H(1) and O(2) is 2.188 and 1.987 Å, respectively. Compared with the tinidazole molecule, the bond lengths of C(2)–N(3) and C(2)–N(1) in M2* are drawn to 1.509 and 1.559 Å, respectively. In addition, the O(1) atom loses a proton, and the O(1)–C(2) bond length is shortened to 1.326 Å, which tends to become a double bond. In TS4*, the C(2)–N(1) bond begins to break and the bond length is 2.265 Å, and the N(3)–C(2) and C(2)–O(1) bond lengths are shortened to 1.443 and 1.227 Å, respectively. It indicates that the covalent bond between N(3) and C(2) and the double bond between C(2) and O(1) is formed gradually. The H(1) and O(1) atoms are adsorbed on the surface of TiO_2_, the distances between H(1) and O(2), O(1) and Ti(5) are 1.957 and 2.198 Å. The activation energy of 14.79 kcal mol^−1^ for TS4*, a unique imaginary frequency of −263 i cm^−1^, and the direction of vibration is correctly directed to the reactants and products. Finally, P* is formed by TS4*, C(2)–N(1) is completely broken, and the distance between them is 3.177 Å (Fig. 6). At the same time, C(2)–N(3) and C(2)–O(1) are also completely formed, and the bond lengths are 1.382 and 1.248 Å, respectively.

We studied the ring-opening reaction mechanism of tinidazole on anatase TiO_2_(101) and (001) surfaces. It was found that the ring-opening reaction can be carried out along the reaction route II on the TiO_2_(101) and (001) surfaces. While compared with the TiO_2_(101) surface, the activation energy of ring-opening degradation on TiO_2_(101) surface is lower, indicating the catalytic activity of (101) surface is stronger. In the ring-opening reaction, the activation energy for the step of bond cleavage is lower, while the proton transfer is reversed. From our research: anatase TiO_2_(101) and (001) surfaces can be used to catalyze the ring opening of tinidazole under the thermal reaction conditions, and anatase TiO_2_ can degrade the tinidazole very well.^53^

In order to investigate the solvent effect, we studied the most stable aqueous solution of B1 and b2 as the reaction starting materials, and studied the effect of solvent effect on the degradation mechanism. And we focused on the ring-opening reaction pathways II, which is proved to be the optimal reaction pathway under gas phase conditions. For TiO_2_(101) surface, the reaction pathway is: R′ → TS3′ → M2′ → TS4′ → P′ (Fig. S8, ESI†). In R′, the tinidazole molecule is adsorbed on TiO_2_ and the distance between the N(3) and the Ti(5) atoms is 2.302 Å. At the same time, ·OH attack C(2) atom, C(2)–O(1) bond length is 1.376 Å, the distance between H(1) atoms and O(2) atoms on the surface of TiO_2_ is 1.749 Å. The bond length of C(2)–N(1) and N(3)–C(2) are extended to 1.536 and 1.475 Å respectively, indicating that the double bond N(3)–C(2) becomes a single bond, at which point the C(2) atom is sp^3^ hybridized. These two bonds are significantly longer than the other covalent bonds on the imidazole ring, and the C(2)–N(1) bond is slightly longer than N(3)–C(2) bond, indicating that the C(2)–N(1) bond is most likely to break. The process R′ → TS3′ is the rate-determining step of the reaction path II, from the process of R → TS3′, H(1) begins to separate from O(1) and transfer to the N(3) atom, and forms a four-membered ring transition state. The distance between H(1) and O(1) atoms, H(1) and N(3) atoms are 2.398, 2.388 Å, and the O(1)–H(1) bond is elongated. The N(3) is separated from Ti(5), O(1) is adsorbed onto the surface of TiO_2_, and the distance from Ti(5) is 2.236 Å. The activation energy for forming TS3′ is 16.07 kcal mol^−1^, and the unique imaginary frequency was −1235 i cm^−1^. In intermediate M2′, H(1) has been transferred to the N(3) atom, and the H(1)–N(3) bond length is 1.045 Å, and the distances between O(1) and Ti(5), H(1) and O(2) are 2.019 and 1.945 Å. In TS4′, the C(2)–N(1) bond begins to break and the bond length is extended to 2.115 Å. The N(3)–C(2), C(2)–O(1) bond lengths are shortened to 1.475 and 1.247 Å, indicating that the covalent bond between N(3) and C(2) and the double bond between C(2) and O(1) gradually formed. The H(1) and O(1) atoms are adsorbed on the surface of TiO_2_, and double adsorption occurs between H(1) and two O(2) atoms, and the adsorption distance is 2.399 and 2.178 Å, respectively. The O(1) atom adsorbed on Ti(5) atom, and the distance between them is 2.107 Å. This step forms an activation energy of 4.42 kcal mol^−1^ for TS4′ and a unique imaginary frequency of −129 i cm ^−1^. Finally, P′ is formed by TS4′, C(2)–N(1) is completely broken, and the distance between them is 3.203 Å. At the same time, the C(2)–N(3) bond and C(2)–O(1) are also completely formed, and the bond lengths are 1.382 and 1.258 Å, respectively. The distances between H(1) and O(2), O(1) and Ti(5) are 1.766 and 2.127 Å, respectively.

For TiO_2_(001) surface, the reaction pathway is: R*′ → TS3*′ → M2*′ → TS4*′ → P*′ (Fig. S9, ESI†). When tinidazole is adsorbed on the TiO_2_(001) surface, in the R*′, the tinidazole molecule is adsorbed on the TiO_2_, and the distance between the N(3) atom and the Ti(5) atom is 1.534 Å. At the same time, the ·OH radical attacks the C(2) atom, the C(2)–O(1) bond length is 1.393 Å, and the distance between the H(1) and the O(2) atoms is 1.809 Å. The C(2)–N(1) and C(2)–N(3) bond lengths are increased from the original 1.370 and 1.332 Å to 1.515 and 1.474 Å, respectively, where the C(2) atom is sp^3^ hybrid. From the bond length analysis, C(2)–N(1), N(3)–C(2) are significantly longer than other covalent bonds on the imidazole ring, indicating that these two bonds may not be as strong as other bonds. The C(2)–N(1) bond is slightly longer than the N(3)–C(2) bond, so the C(2)–N(1) is the most likely to break and ring-opening. From the process of R*′ → TS3*′, H(1) begins to separate from O(1) and transfer to the N(3) atom to form a four-membered ring transition state. The distances between H(1) and O(1), H(1) and N(3) atoms are 1.508 and 1.867 Å, respectively, and the O(1)–H(1) bond is elongated. The activation energy for forming TS3*′ is 35.23 kcal mol^−1^, with a unique imaginary frequency of −1263 i cm^−1^, and the direction of vibration is correctly directed to the reactants and products. The process R*′ → TS3*′ is the control step of the reaction path II. In M2*′, H(1) atom has been transferred to the N(3) atom and the H(1)–N(3) bond length is 1.035 Å. The O(1) and H(1) atoms are attracted to the surface of TiO_2_, the distances between O(1) and Ti(5), H(1) and O(2) are 2.142 and 1.959 Å. Compared with the tinidazole molecule, the bond lengths of C(2)–N(3) and C(2)–N(1) in M2*′ were elongated to 1.515 and 1.566 Å. In addition, O(1) atom loses a proton, and the O(1)–C(2) bond length is shortened to 1.321 Å, which has a tendency to form a double bond. In TS4*′, the C(2)–N(1) bond begins to break, and the bond length is extended to 2.265 Å. The N(3)–C(2), C(2)–O(1) bond lengths were shortened to 1.443 and 1.265 Å, respectively, indicating a covalent bond between N(3) and C(2) and a double bond between C(2) and O(1) gradually formed. H(1) and O(1) atoms adsorbed on the surface of TiO_2_, the distances between H(1) and O(2), O(1) and Ti(5) are 1.957 and 2.194 Å, respectively. This step forms an activation energy of 14.76 kcal mol^−1^ for TS4*′, a unique imaginary frequency of −256 i cm^−1^, and the direction of vibration is correctly directed to the reactants and products. Finally, P*′ is formed by TS4*′, C(2)–N(1) is completely broken, and the distance between them is 3.165 Å. At the same time, the C(2)–N(3) bond and C(2)–O(1) are also completely formed, and the bond lengths are 1.387 and 1.250 Å. The distance between H(1) and O(2), O(1) and Ti(5) is 1.998 and 2.292 Å, respectively. Under the condition of aqueous solvent model, the ring-opening reaction mechanism of tinidazole on TiO_2_(101) and (001) surfaces is the same as that in the gas phase (Tables S2 and S4, ESI†). But the activation energy of the reaction changes, in the water solvent model, the activation energy of the degradation reaction is reduced.

All our previous calculations are conducted using PBE functional alone using DMol^3^ module that implemented in MS. However, it becomes more and more important in recent years to include the DFT+U and van der Waals corrections in DFT calculations to obtain more reliable results.^54–56^ In order to evaluate whether such corrections have significant influences to our present conclusions, we have employed the Vienna *ab initio* simulation package (VASP)^57–61^ to calculate the single point energies based on the adsorption configurations and reaction paths optimized by DMol^3^ with the DFT+U^62^ (U corrections was applied to the d-orbital of Ti atom with a value of 4.0 eV) and van der Waals corrections.^63,64^ The corrected results are listed in Table S5 in ESI.† Although there are some differences about the exact energies of these structures, the results are in qualitatively agreement with previous results, *i.e.* A5, a5, B1 and b2 are the most stable adsorption configurations with the highest adsorption energy of 2.04, 2.76, 4.06 and 11.13 eV. As for the mechanism of the ring-opening degradation reaction, as shown in Table S6 of ESI,† the mechanism of reaction pathway I is R → TS1 → M1 → TS2 → P for the TiO_2_(101) surface, the activation energy of TS3 and TS4 are 37.51 and 70.82 kcal mol^−1^, respectively. And the mechanism of reaction pathway II is R → TS3 → M2 → TS4 → P for the TiO_2_(101) surface, the activation energy of TS3 and TS4 are 29.54 and 21.34 kcal mol^−1^, respectively. Obviously, the energy barrier of path II is lower than that of path I, which means that path II is better. For the TiO_2_(001) surface, the activation energy of TS1* and TS2* are 23.11 and 61.33 kcal mol^−1^, respectively. And the activation energy of TS3* and TS4* are 44.64 and 12.83 kcal mol^−1^, respectively. It also means that path II is better. By comparing the (001) and (101) surface, we can also conclude that the TiO_2_(101) surface is better. Although the energy barrier calculated by VASP is somewhat larger than that by MS, the same conclusion can be drawn by our present calculations after the DFT+U and van der Waals corrections, *i.e.* the ring-opening along the reaction path II on the (101) surface is the most probable reaction pathways. In our subsequent investigations, we will include the DFT+U and van der Waals corrections directly in our optimizations to provide more solid conclusions.

## 4. Conclusions

In summary, we have studied the adsorption characteristics and degradation mechanism of tinidazole on the anatase TiO_2_(101) and (001) surfaces under vacuum and solvation conditions. It showed that the most stable adsorption configuration was the N(3) atom adsorbed on the Ti(5) atom of TiO_2_ with the largest adsorption energy. The adsorption makes the bond length of C–N longer, which is beneficial to the ·OH radicals attack and ring-opening reaction. The density states of the adsorption configuration, the utilization efficiency of two surfaces of anatase TiO_2_ in the visible light, and predicted the photocatalysis were obtained. In the mechanism of ring-opening degradation reaction, it was found that the ring opening site was in the C–N. The ring-opening reaction can be carried out along the reaction route II on the TiO_2_(101) and (001) surfaces, and the reaction activate energy is lower on (101) surface. By studying the effect of water solvation on the reaction, it is found that the activation energy of tinidazole in the degradation of TiO_2_ crystal surface is reduced, indicating that the water solvent can promote the degradation reaction.

## Conflicts of interest

There are no conflicts to declare.

## Supplementary Material

RA-010-C9RA06665A-s001
