# Supplementary material for: The adsorption characteristics and degradation mechanism of tinidazole on an anatase TiO2 surface: a DFT study
Source: RSC Adv. 2020 Jan 10;10(4):2104–12. doi: 10.1039/c9ra06665a (PMC9048433; doi:10.1039/c9ra06665a)
Supplement: RA-010-C9RA06665A-s001 [file RA-010-C9RA06665A-s001.pdf]

Electronic Supplementary Material (ESI) for RSC Advances.
This journal is © The Royal Society of Chemistry 2020

The adsorption characteristics and degradation mechanism of tinidazole on anatase TiO_2 surface: A DFT study

Qiaoqiao Qin[1], Haichuan Qin[1] ,Kai Li[1], Ruolan Tan[2] ,Xiangyang Liu[1], and Laicai Li[1*]

[1] College of Chemistry and Material Science, Sichuan Normal University, Chengdu 610068, China.

[2] College of Pharmacy, Southwestern Medical University, Luzhou, 646000, China.

Content:

Fig. S1 The adsorption configurations and adsorption distances (Å) of tinidazole on TiO_2(101) and (001) surfaces under aqueous conditions(Front view and Top view)

Fig. S2 The DOSs and PDOSs of TiO_2(101) surface and tinidazole adsorption configurations in vacuum conditions

Fig. S3 The DOSs and PDOSs of TiO_2(001) surface and tinidazole adsorption configurations in vacuum conditions

Fig. S4 The DOSs and PDOSs of TiO_2(101) surface and tinidazole adsorption configurations in aqueous conditions

Fig. S5 The DOSs and PDOSs of TiO_2(001) surface and tinidazole adsorption configurations in aqueous conditions

Fig. S6 The configuration of substances in the reaction of tinidazole on the TiO_2(101) surface in vacuum conditions (Top view)

Fig. S7 The configuration of substances in the reaction of tinidazole on the TiO_2(001) surface in vacuum conditions (Top view)

Fig. S8 The configuration of substances in the reaction of tinidazole on the TiO_2(101) surface in aqueous conditions (Front view and Top view)

Fig. S9 The configuration of substances in the reaction of tinidazole on the TiO_2(001) surface in aqueous conditions (Front view and Top view)

Table S1. The change of the bond length of substances in the reaction of tinidazole on TiO_2(101) surface

Table S2 Energy and frequency of substances in the reaction of tinidazole on TiO_2(101) surface

Table S3 The change of the bond length of substances in the reaction of tinidazole on TiO_2(001) surface

Table S4 Energy and frequency of substances in the reaction of TNZ on TiO_2 (001) surface

Table S5 Adsorption energies for adsorption configurations of tinidazole adsorbed on TiO_2(101) and (001) surfaces in the VASP program

Table S6 Energy of substances in the reaction of tinidazole on TiO_2 in the VASP program

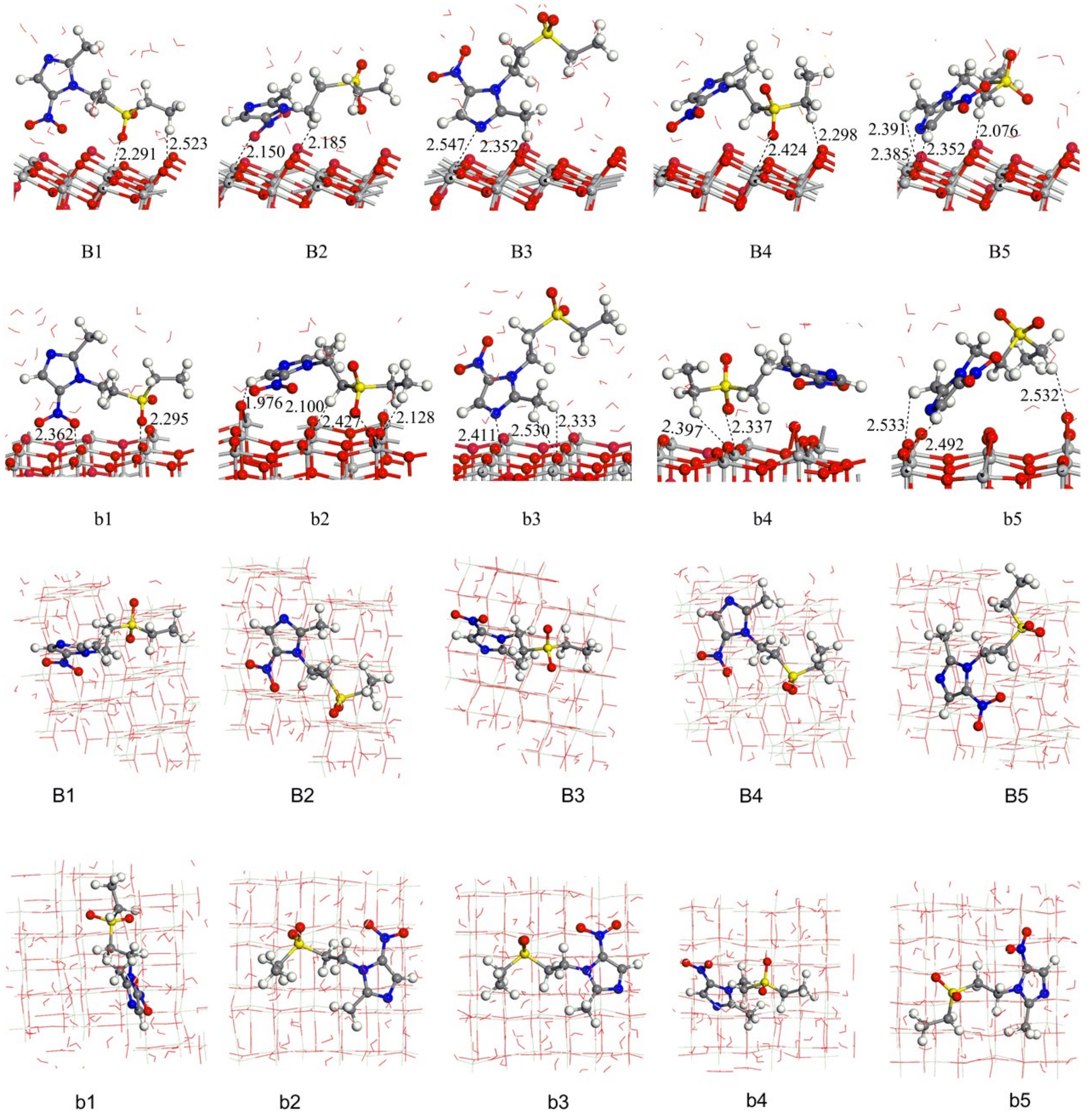

Fig. S1 The adsorption configurations and adsorption distances (Å) of tinidazole on TiO_2(101) and (001) surfaces under aqueous conditions(Front view and Top view)

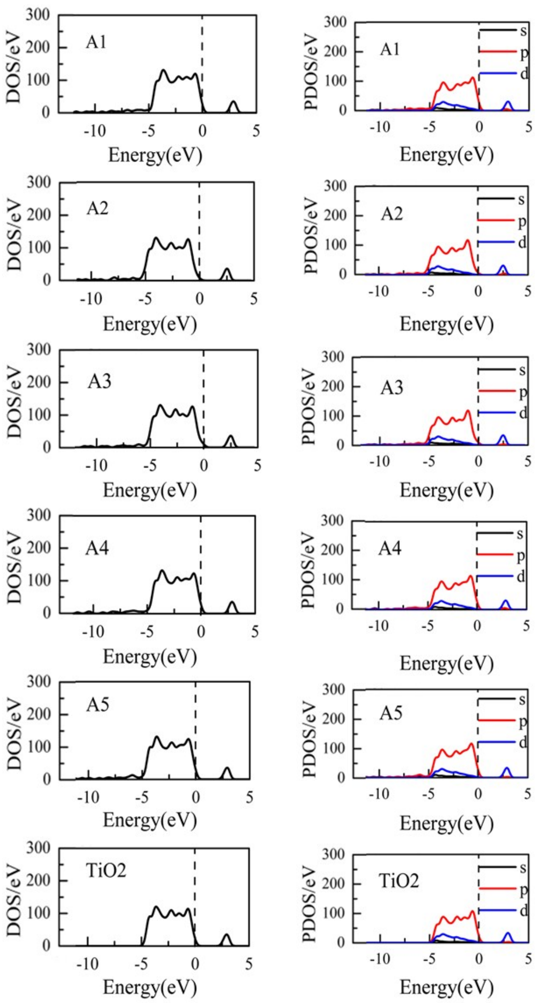

Fig. S2 The DOSs and PDOSs of TiO_2(101) surface and tinidazole adsorption configurations in vacuum conditions

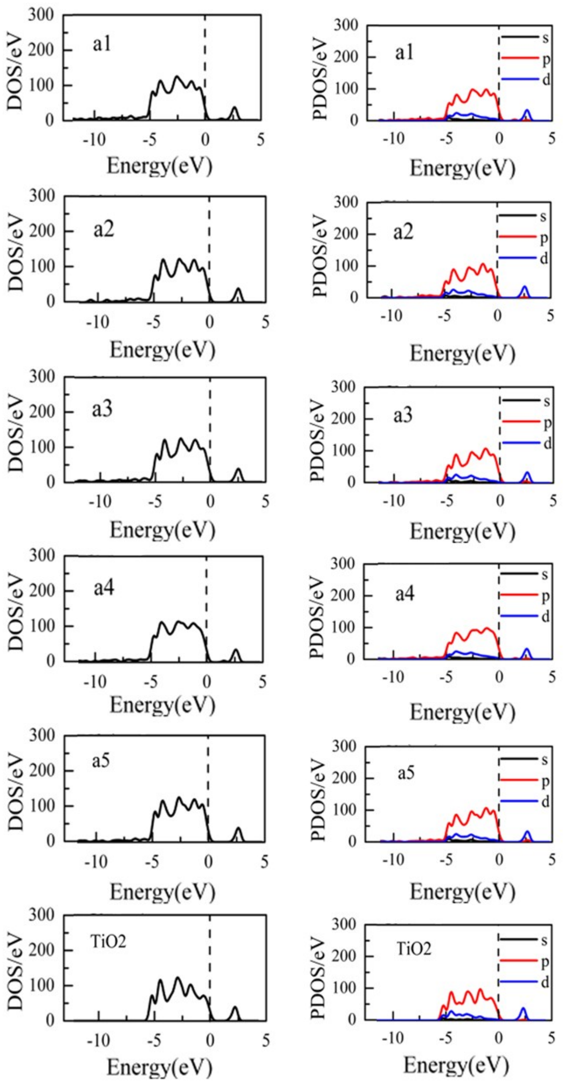

Fig. S3 The DOSs and PDOSs of TiO_2(001) surface and tinidazole adsorption configurations in vacuum conditions

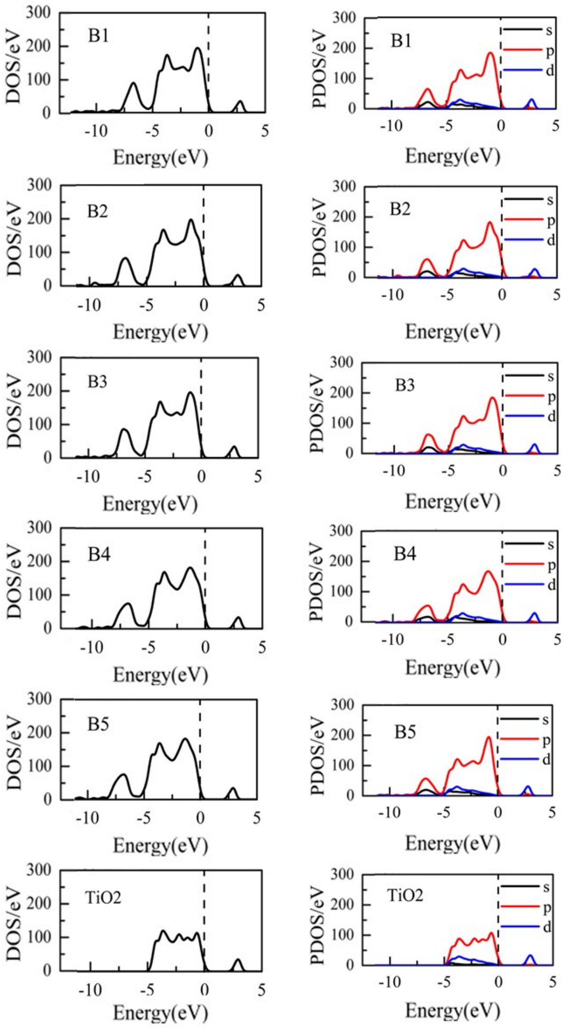

Fig. S4 The DOSs and PDOSs of TiO_2(101) surface and tinidazole adsorption configurations in aqueous conditions

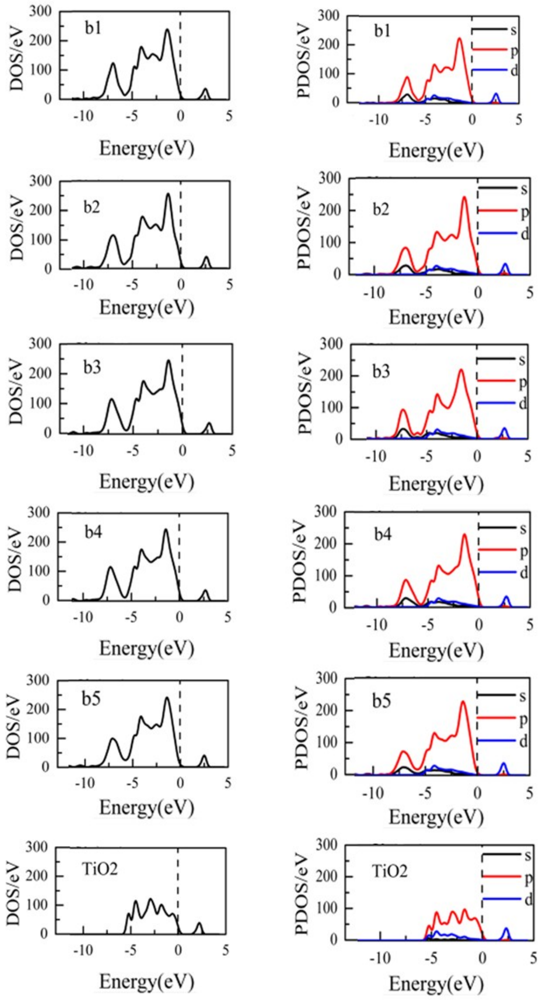

Fig. S5 The DOSs and PDOSs of TiO_2(001) surface and tinidazole adsorption configurations in aqueous conditions

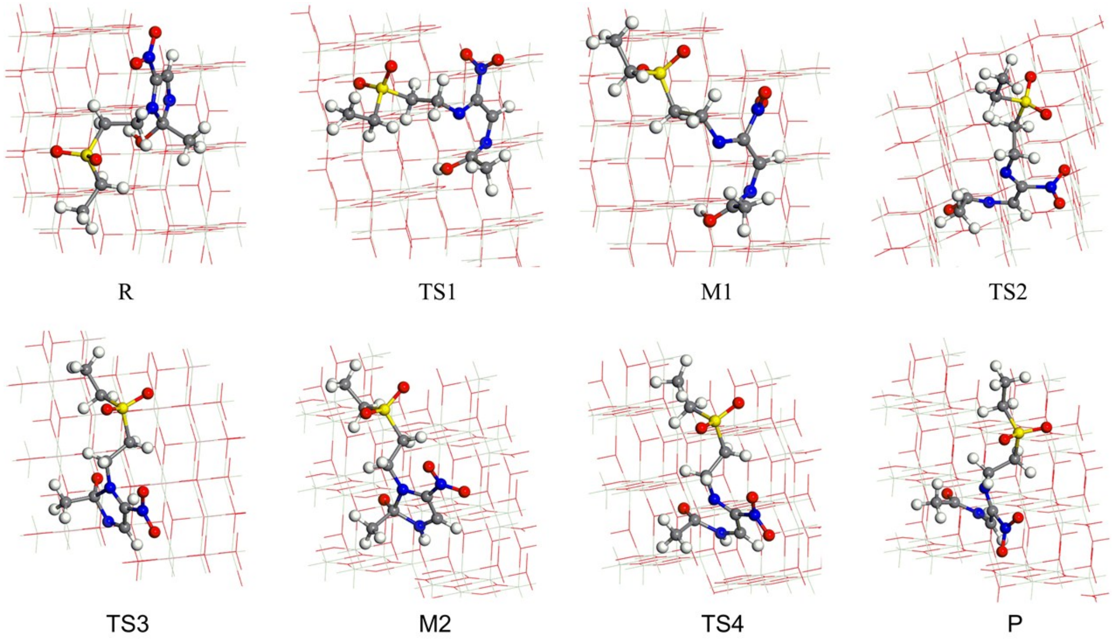

Fig. S6 The configuration of substances in the reaction of tinidazole on the TiO_2(101) surface in vacuum conditions (Top view)

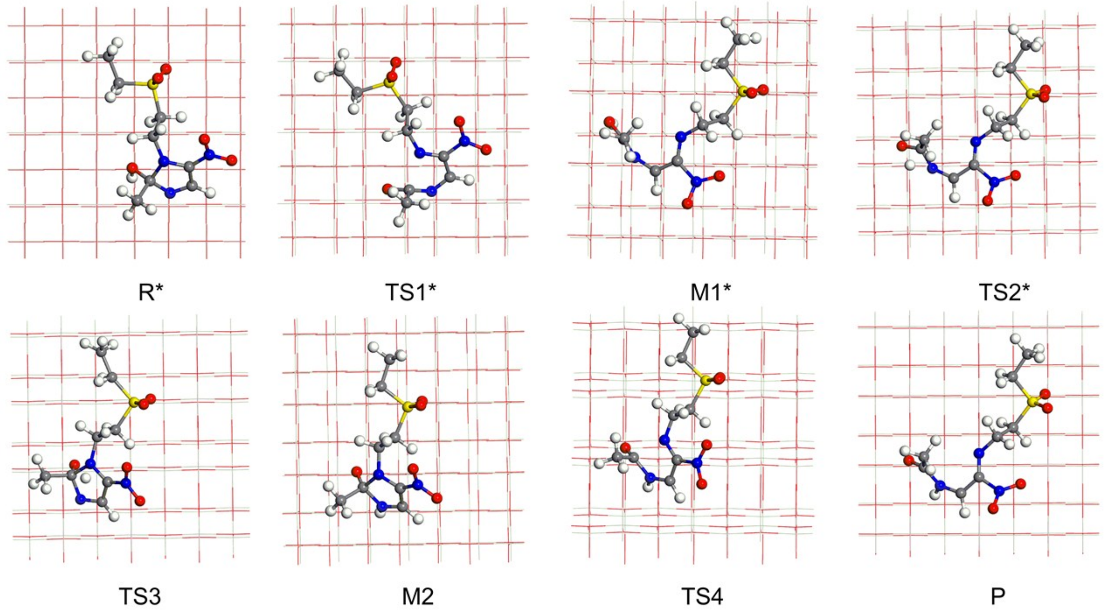

Fig. S7 The configuration of substances in the reaction of tinidazole on the TiO_2(001) surface in vacuum conditions (Top view)

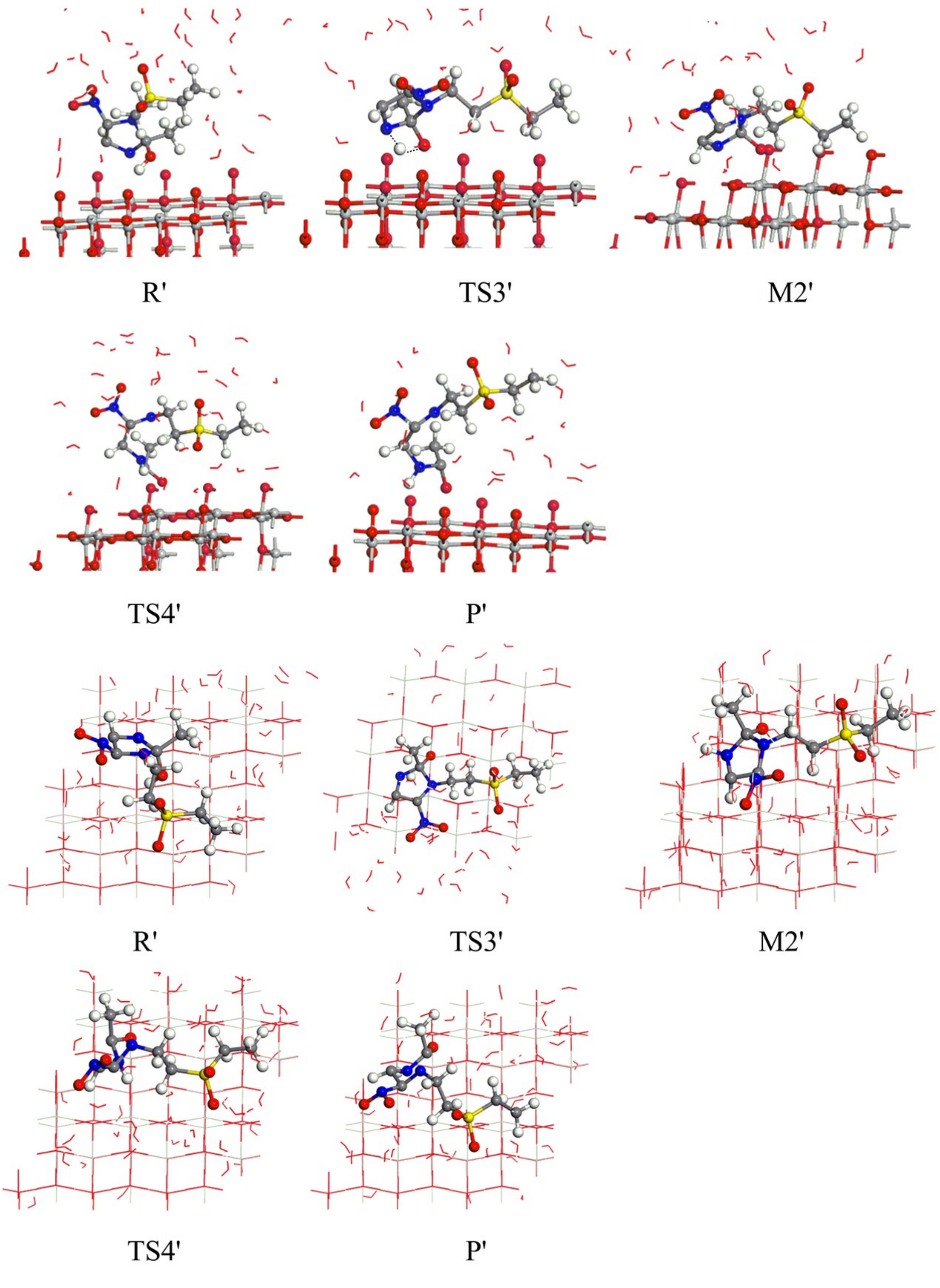

Fig. S8 The configuration of substances in the reaction of tinidazole on the TiO_2(101) surface in aqueous conditions (Front view and Top view)

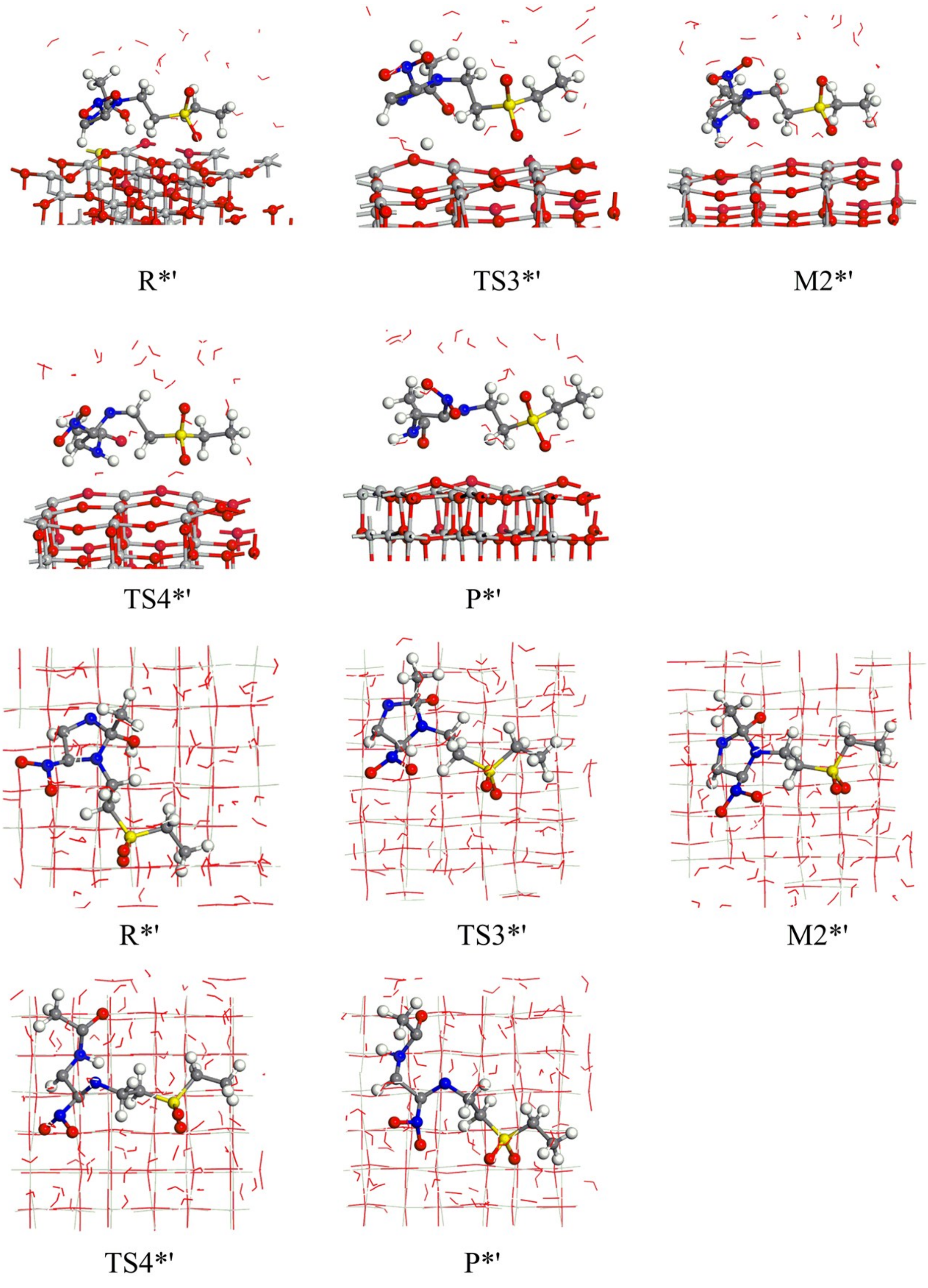

Fig. S9 The configuration of substances in the reaction of tinidazole on the TiO_2(001) surface in aqueous conditions (Front view and Top view)

Table S1. The change of the bond length of substances in the reaction of tinidazole on TiO_2(101) surface

Pathway	Compound	Bond length(Å)					
		N(1)-C(2)	C(2)-N(3)	N(3)-C(4)	C(2)-O(1)	O(1)-H(1)	N(3)-H(1)
I	R	1.525	1.470	1.312	1.388	1.016	—
	TS1	2.345	1.358	1.313	1.307	1.096	—
	M1	2.944	1.390	1.323	1.247	1.154	—
	TS2	2.794	1.303	1.304	1.308	1.207	2.158
	P	3.206	1.372	1.373	1.258	—	1.053
II	TS3	1.682	1.469	1.228	1.326	2.237	2.236
	M2	1.552	1.520	1.320	1.323	—	1.042
	TS4	2.201	1.462	1.329	1.240	—	1.133

Table S2 Energy and frequency of substances in the reaction of tinidazole on TiO_2(101) surface

pathway	Compound	E（hatree）	E_r(Kcal mol^{-1})	V(i cm^{-1})	Compound	E（hatree）	E_r(Kcal mol^{-1})	v(i cm^{-1})
I	R	-8772.4742057	0		R′	-8772.5485934	0	
	TS1	-8772.4285793	28.63	-260	TS1′	-8772.4992772	30.95	-290
	M1	-8772.5013747	-17.05		M1′	-8772.5668226	-11.44	
	TS2	-8772.4191941	34.52	-1141	TS2′	-8772.5944588	28.78	-1684
	P	-8772.4683927	3.65		P′	-8772.5525182	-2.46	
II	TS3	-8772.4400441	21.44	-1646	TS3′	-8772.5229804	16.07	-1601
	M2	-8772.4705240	2.31		M2′	-8772.5404442	5.11	
	TS4	-8772.4484657	16.15	-129	TS4′	-8772.5333028	9.59	-257

Table S3 The change of the bond length of substances in the reaction of tinidazole on TiO_2(001) surface

Pathway	Compound	Bond length(Å)					
		N(1)-C(2)	C(2)-N(3)	N(3)-C(4)	C(2)-O(1)	O(1)-H(1)	N(3)-H(1)
I	R*	1.519	1.473	1.312	1.397	1.016	—
	TS1*	2.199	1.396	1.322	1.297	1.038	—
	M1*	3.043	1.330	1.340	1.308	1.073	—
	TS2*	3.055	1.345	1.309	1.302	1.237	1.467
	P*	3.177	1.382	1.373	1.248	—	1.046
II	TS3*	1.579	1.465	1.315	1.434	1.612	1.816
	M2*	1.559	1.509	1.331	1.326	—	1.035
	TS4*	2.265	1.443	1.344	1.227	—	1.042

Table S4 Energy and frequency of substances in the reaction of TNZ on TiO_2 (001) surface

pathway	Compound	E（hatree）	E_r(Kcal mol^{-1})	V(i cm^{-1})	Compound	E（hatree）	E_r(Kcal mol^{-1})	v(i cm^{-1})
I	R*	-8772.4551550	0		R*′	-8772.5090559	0	
	TS1*	-8772.4221747	20.70	-264	TS1*’	-8772.4728783	22.70	-287
	M1*	-8772.4532121	1.22		M1*′	-8772.5014883	4.75	
	TS2*	-8772.3607533	59.24	-1549	TS2*′	-8772.4140780	59.60	-1656
	P*	-8772.4575051	-1.47		P*′	-8772.5146659	-3.52	
II	TS3*	-8772.3927960	39.13	-1265	TS3*′	-8772.5229804	35.23	-1263
	M2*	-8772.4475535	4.77		M2*′	-8772.5404442	1.34	
	TS4*	-8772.4239858	19.56	-263	TS4*′	-8772.5333028	16.10	-256

Table S5 Adsorption energies for adsorption configurations of tinidazole adsorbed on TiO_2 (101) and (001) surfaces in the VASP program

	(101) surface		(001) surface	
conditions	compound	E_{ads} (eV)	compound	E_{ads}(eV)
Vacuum conditions	A1	1.82	a1	2.60
	A2	1.59	a2	2.68
	A3	1.54	a3	2.48
	A4	0.75	a4	2.62
	A5	2.04	a5	2.76
Aqueous solutions	B1	4.06	b1	3.97
	B2	-11.15	b2	11.13
	B3	3.44	b3	3.90
	B4	3.78	b4	3.86
	B5	3.85	b5	3.45

Table S6 Energy of substances in the reaction of tinidazole on TiO_2 in the VASP program

(101) surface				(001) surface			
Compound	E(eV)	E_r(Kcal mol^{-1})	E_a(Kcal mol^{-1})	Compound	E(eV)	E_r(Kcal mol^{-1})	E_a(Kcal mol^{-1})
R	-1012.05690	0.00		R*	-1010.46510	0.00	
TS1	-1010.43050	37.51	37.51	TS1*	-1009.46280	23.11	23.11
M1	-1013.05110	-22.93		M1*	-1010.47520	-0.23	
TS2	-1009.97870	47.92	70.85	TS2*	-1007.81560	61.10	61.33
TS3	-1010.77580	29.54	29.54	TS3*	-1008.52920	44.64	44.64
M2	-1012.02240	0.80		M2*	-1009.98810	11.00	
TS4	-1011.09700	22.14	21.34	TS4*	-1009.43160	23.83	12.83
P	-1011.56210	11.41		P*	-1010.34660	2.73	
R1'	-1017.09940	0.00		R1*'	-1013.52880	0.00	
TS3'	-1016.31600	18.07	18.07	TS3*'	-1011.84460	38.84	38.84
M2'	-1016.76680	7.67		M2*'	-1013.19300	7.74	
TS4'	-1016.23420	19.95	12.28	TS4*'	-1012.71710	18.72	10.98
P'	-1016.69930	9.23		P*'	-1013.50350	0.58	
